# Mitochondria in Sex Hormone-Induced Disorder of Energy Metabolism in Males and Females

**DOI:** 10.3389/fendo.2021.749451

**Published:** 2021-12-20

**Authors:** Lijun Yin, Man Luo, Ru Wang, Jianping Ye, Xiaohui Wang

**Affiliations:** ^1^ School of Kinesiology, Shanghai University of Sport, Shanghai, China; ^2^ Metabolism Research Center, Zhengzhou University Affiliated Zhengzhou Central Hospital, Zhengzhou, China; ^3^ Center for Advanced Medicine, College of Medicine, Zhengzhou University, Zhengzhou, China; ^4^ Shanghai Diabetes Institute, Shanghai Jiao Tong University Affiliated Sixth People’s Hospital, Shanghai, China

**Keywords:** mitochondria, sex hormone, energy metabolism, insulin sensitivity, sex dimorphism

## Abstract

Androgens have a complex role in the regulation of insulin sensitivity in the pathogenesis of type 2 diabetes. In male subjects, a reduction in androgens increases the risk for insulin resistance, which is improved by androgen injections. However, in female subjects with polycystic ovary syndrome (PCOS), androgen excess becomes a risk factor for insulin resistance. The exact mechanism underlying the complex activities of androgens remains unknown. In this review, a hormone synergy-based view is proposed for understanding this complexity. Mitochondrial overactivation by substrate influx is a mechanism of insulin resistance in obesity. This concept may apply to the androgen-induced insulin resistance in PCOS. Androgens and estrogens both exhibit activities in the induction of mitochondrial oxidative phosphorylation. The two hormones may synergize in mitochondria to induce overproduction of ATP. ATP surplus in the pancreatic β-cells and α-cells causes excess secretion of insulin and glucagon, respectively, leading to peripheral insulin resistance in the early phase of type 2 diabetes. In the skeletal muscle and liver, the ATP surplus contributes to insulin resistance through suppression of AMPK and activation of mTOR. Consistent ATP surplus leads to mitochondrial dysfunction as a consequence of mitophagy inhibition, which provides a potential mechanism for mitochondrial dysfunction in β-cells and brown adipocytes in PCOS. The hormone synergy-based view provides a basis for the overactivation and dysfunction of mitochondria in PCOS-associated type 2 diabetes. The molecular mechanism for the synergy is discussed in this review with a focus on transcriptional regulation. This view suggests a unifying mechanism for the distinct metabolic roles of androgens in the control of insulin action in men with hypogonadism and women with PCOS.

## Introduction

Testosterone is the primary sex hormone in regulating male sex organ development and reproduction activities. It is produced by the testis in males and ovarian interstitial cells in females. Testosterone is converted into the more active form, dihydrotestosterone (DHT), in the cell cytoplasm by 5α-reductase. DHT directly activates the androgen receptor (AR) for induction of target gene transcription. After DHT binds to its receptor, the AR undergoes a conformational change to disassociate from the heat shock protein and travels to the nucleus. In the nucleus, AR binds to the androgen response elements (AREs) of target genes to modulate gene transcription for the control of male organ development, reproductive cell differentiation, muscle growth, bone strength, and acceleration of energy (glucose, fatty acids, and amino acids) metabolism. Additionally, androgens have receptor-independent activity in the regulation of endothelial cell proliferation (1). Testosterone-driven energy metabolism favors energy expenditure to prevent metabolic disorders, such as obesity and type 2 diabetes in male subjects (2, 3). Testosterone deficiency (hypogonadism) increases the risk of metabolic disorders in male subjects and animals (4–6). In some cases, however, hypogonadism can be the result of obesity due to hypothalamus–pituitary axis inhibition by aromatase conversion of androgen into estradiol in peripheral tissues [such as white adipose tissue (WAT)] (7). Injection of male hormones is a treatment strategy for obesity and type 2 diabetes in male patients with hypogonadism. Although the hormone treatment is effective in most studies, there are reports of inefficacy in some studies (8, 9).

In female subjects, testosterone excess (hypergonadism) is a risk factor for type 2 diabetes due to testosterone’s role in the induction of insulin resistance (10). Elevated plasma testosterone levels are often associated with insulin resistance in obese girls (11) and women with polycystic ovary syndrome (PCOS) (12, 13). Inhibition of testosterone production and antagonism of its activity are clinical strategies to control insulin resistance in PCOS patients (14–16). Treatment often leads to visceral fat reduction and improved insulin sensitivity. Studies have shown that testosterone exhibits opposite effects on the regulation of insulin sensitivity in male versus female patients (17, 18); however, the exact mechanism remains unknown. Here, we propose a potential mechanism by integration of multidisciplinary information with a focus on mitochondria to explain the androgen activity. Estrogen protects insulin sensitivity in female subjects (19). Reduced estrogen levels in postmenopausal patients lead to an increased risk of insulin resistance, which can be improved by estrogen supplementation (19). Both estrogens and androgens regulate metabolism through nuclear receptors. Activation of these receptors involves transcription-mediated reprogramming of the neuroendocrine system according to current studies; however, there is no unifying mechanism of how sex hormones regulate insulin sensitivity in PCOS. Mitochondria are the targets of both hormones (more discussion below). Interestingly, mitochondrial genes that play a role in oxidative phosphorylation and antioxidant properties have been observed to be sexually dimorphic in the skeletal muscle and liver of female rats (20). Therefore, we propose that mitochondria may be the key targets for androgens and estrogens in the control of insulin sensitivity, which may hold an answer for the gender-dependent effects of androgens. This review will integrate information from several fields, such as obesity, diabetes, cardiovascular diseases, endocrinology, and mitochondrial biology to explore the mechanism. The aim is to propose a mitochondrion-centered mechanism for how androgens together with estrogen regulate insulin sensitivity, with energy metabolism in mitochondria as the primary focus.

## Mitochondria in Energy Expenditure

Mitochondria are the center of energy metabolism in cells, as mitochondria carry out both the catabolism and anabolism of substrates for fuel (21). In the catabolism process, substrates such as glucose, fatty acids, and amino acids are broken down to generate ATP or heat through OXPHOS, which is used as energy for cellular activities. Heat production (thermogenesis) is required for the maintenance of body temperature in mammalians (22). Induction of thermogenesis is an ideal approach in the control of obesity. Thermogenesis includes UCP1-dependent and UCP1-independent mechanisms (23). The UCP1-dependent mechanism is dominant in brown and beige adipocytes (24). The UCP1-independent system comprises the adenine nucleotide transporters 1/2 (ANT1/2) and UCP3, which play a major role in non-adipocytes (23). Mammalian ANT is a “new” uncoupling protein found in the mitochondria of several tissues including the muscle, kidney, liver, and brown fat (25) and is equivalent to the ADP/ATP carrier (AAC) in yeast. The reduction of energy output by dysfunctional mitochondrial may lead to a buildup of intermediate metabolites, which plays a role in the pathogenesis of insulin resistance.

Mitochondria export intermediate metabolites, such as acetyl-CoA and oxaloacetate to use for anabolism. In the cytoplasm, acetyl-CoA is a substrate for *de novo* lipogenesis of fatty acids and cholesterols. In hepatocytes, oxaloacetate is a substrate for gluconeogenesis. Insulin plays a role in regulating these metabolite levels by stimulating lipogenesis and inhibiting gluconeogenesis. However, these metabolites may also feedback to impact insulin sensitivity. In the lipotoxicity model of insulin resistance, acetyl-CoA buildup inhibits insulin-induced glucose utilization in the skeletal muscle through substrate competition (26). Oxaloacetate buildup in liver hepatocytes promotes gluconeogenesis, contributing to insulin resistance of the liver. In obesity, overproduction of these metabolites in tissues contributes to systemic insulin resistance, and mitochondrial dysfunction in OXPHOS results in insulin resistance through the buildup of the metabolites (27).

Mitochondrial function is regulated by multiple factors, such as biogenesis and mitophagy. Each mitochondrion contains about 1,200 different types of proteins, of which 13 proteins are encoded by mitochondrial genome DNA (mtDNA), and the rest are encoded by nuclear DNA (28). Crosstalk of the nuclear and mitochondrial genomes is required for mitochondrial biogenesis. In the mechanism, the biogenesis is controlled by a network of transcription factors, such as peroxisome proliferator-activated receptor γ (PPARγ), PPARα (29, 30), estrogen-related receptors (ERRs) (31), cAMP response element-binding protein (CREB), and Forkhead box transcription factor (FOXO) (32, 33). In addition, these transcription factors require peroxisome proliferator-activated receptor γ coactivator 1 alpha (PGC-1α) as a primary cofactor. They induce expression of other transcription factors, such as nuclear respiratory factor-1 (NRF-1), which in turn activates expression of mitochondrial DNA transcription factor A (TFAM) in the nucleus (34–36). TFAM is transferred into mitochondria to induce expression and duplication of mtDNA (37, 38). TFAM also requires PGC-1α in the induction of mitochondrion-related gene expression (39). NRF-2, an isoform of NRF-1, is required for expression of the cytochrome *c* oxidase (COX) in complex IV of the respiratory chain, in which NRF-2 interacts with PGC-1α (30). Therefore, PGC-1α is a major coactivator in the transcription network for mitochondrial biogenesis. Hormones that induce the PGC-1α activity may induce mitochondrial biogenesis through this transcription network.

Mitophagy is the process by which mitochondria are recycled. Mitochondrial components are frequently damaged by high levels of reactive oxygen species (ROS). ROS levels are increased upon active ATP production and have been shown to be coupled with heat production (40, 41). Irreversibly damaged mitochondria are removed by the process of mitophagy (42), a specific form of autophagy in the quality control system of mitochondria. Mitophagy is regulated by PTEN-induced kinase 1 (PINK1), which is on the outer mitochondrial membrane (OMM). In the damaged mitochondria, PINK1 recruits Parkin through phosphorylation. Parkin promotes mitophagy through ubiquitination of proteins on the mitochondrial membrane in the formation of autophagosomes (43). Other mitochondrial proteins, such as NIX, BNIP3, and FUNDC1, are also involved in mitophagy, and a defect in any of those molecules may contribute to impaired mitophagy. A defect in mitophagy has been reported in the pathogenesis of insulin resistance in several studies (44–46). Therefore, dysregulation of the mitochondria quality control process may lead to mitochondrial dysfunction in the pathogenesis of insulin resistance.

## Energy Surplus Leads to Mitochondrial Dysfunction in Obesity

Obesity and type 2 diabetes represent the body’s compensatory responses to energy surplus conditions. In these conditions, mitochondria suffer from an oversupply of fuel substrates such as lipids, glucose, amino acids, and their derivatives. A chronic state of fuel surplus may lead to mitochondrial dysfunction and reduction in ATP production capacity (47). Mitochondrial inflexibility is a type of mitochondrial dysfunction in which substrate switch between fatty acids and glucose is disordered (26). This concept, however, is challenged by a new study (48). The signaling mechanism of mitochondrial inflexibility remains unclear (27). We propose that mitochondrial inflexibility is a mitochondrial compensatory response to fuel surplus, where signaling pathways are involved in the suppression of AMPK and Sirtuin-1 (SIRT1) and the activation of mammalian target of rapamycin (mTOR). This mitochondrial dysfunction worsens metabolic disorders by reducing energy expenditure, which leads to further accumulation of fuels in the insulin-sensitive cells (49). These mitochondrial dysfunctions are improved by lifestyle modifications such as physical exercise, calorie restriction, and weight loss, which are established strategies in the control of obesity, type 2 diabetes, and metabolic disorders (27). These practices induce activation of AMPK and SIRT1, while reducing mTOR activity at the molecular level (50). These molecular pathways provide a mechanism for the correction of mitochondrial dysfunction in the practices, suggesting that energy surplus is a major cause of mitochondrial dysfunction.

The activities of AMPK and SIRT1 are reduced in obesity and type 2 diabetes as a result of energy surplus. Energy status in cells is sensed by molecules including AMPK, SIRT1, and mTOR (51). AMPK is activated in energy-deficient states in order to restore energy supply (51, 52). AMPK activity is reduced when there is a rise in ATP or ATP/AMP ratio, which occurs during states of overfeeding, obesity, type 2 diabetes, and lack of physical exercise (51). Inhibition of AMPK leads to mitochondrial degeneration or dysfunction in the energy surplus condition (51). In addition, AMPK inhibition limits the mobilization of energy substrates, leading to an accumulation of glycogen and fatty acids in the cytoplasm (51). AMPK inhibition decreases the phosphorylation of a mitochondrial scaffold protein, a kinase anchor protein 1 (AKAP1), therefore decreasing mitochondrial ATP/heat production through suppression of the respiratory chain (53). AMPK inhibition also induces a reduction in SIRT1 activity (54). A rise in NADH/NAD^+^ ratio, another indicator of energy status, reduces SIRT1 activity as well. In diabetes and obesity, excess insulin, branch chain amino acids, and ATP activate mTOR. This mTOR activation induces downregulation of mitochondrial biogenesis- and autophagy-related genes (50). Taken together, alteration in AMPK, SIRT1, and mTOR activity occur in energy surplus states such as obesity and type 2 diabetes to compensate for mitochondrial dysfunction and metabolic disorders.

## Metabolic Organs Targeted by Testosterone

Testosterone controls energy metabolism through the actions in several organs, including the brain (55), skeletal muscle, adipose tissue (56), liver, and pancreatic islet cells (4, 13, 57, 58). Here, the discussion focuses on the liver for the availability of transcriptomic data. The testosterone activity is mediated by AR activity, which is regulated by post-translational modifications including phosphorylation, acetylation, methylation, SUMOylation, and ubiquitination [more detail in review (59)]. Testosterone controls energy metabolism through the induction of gene transcription, which has been investigated in the liver of castrated male pigs using the RNA-seq strategy. Testosterone deficiency led to reduced gene expression in multiple metabolic pathways, such as fatty acid oxidation, steroid biosynthesis, cholesterol and bile acid metabolism, and glucose metabolism (60). Analysis conducted with the Kyoto Encyclopedia of Genes and Genomes (KEGG) database suggested that energy output by mitochondria is reduced when fatty acid synthesis and tricarboxylic acid (TCA) cycle activity is decreased (60). Decreased mitochondrial energy output led to an upregulation of inflammatory proteins, oxidative stress, and apoptotic responses. This study suggests that testosterone deficiency causes decreased catabolism of energy substrates (glucose and fatty acids) in mitochondria during ATP and heat production. The effects of testosterone may apply to other tissues as well, such as the skeletal muscle, fat, and pancreatic islet cells (61). In testosterone-deficient patients and animals, decreased catabolism of energy substrates increased the risk of obesity. For example, castration of male rats induced expression of fatty acid synthesis-related genes, thereby leading to fat accumulation in the skeletal muscle and increased subcutaneous fat deposition in normal diet-fed rats (58). In the skeletal muscle, androgens promote myogenesis in male mice and humans by enhancing the expression of glycogen synthase, GLUT4, and insulin receptor substrate 1 (IRS1) (19). In β-cell, testosterone has a protective effect because β-cell-specific AR knockout (ARKO) male mice experience glucose intolerance and β-cell failure similar to hypogonadal men (62, 63). Together, these results illustrate the important effects of androgens on energy metabolism. However, the transcriptome of other tissues in testosterone-deficient animals and patients remains to be revealed.

## Testosterone Induces Mitochondrial Biogenesis

Testosterone affects mitochondrial function in several ways, including mitochondrial structure. Mitochondria have two layers of membranes, OMM and inner mitochondrial membrane (IMM). The IMM holds the respiratory chain and maintains the mitochondrial membrane potential by pumping protons into the intermembrane space. The cristae of the IMM provide structural support to the respiratory chain. Under pathological conditions, the orderly arrangement of the tubular and lamellar mitochondrial cristae may be disrupted (64). In androgen-deficient rats, crista number and length are reduced in the cardiomyocytes, but these pathologic changes are reversed by androgen supplementation (65). Similar changes are observed in ARKO mice (66). The mechanism behind androgen’s role in the regulation of cristae has yet to be elucidated and may be related to gene expression. These studies suggest that androgen is required in the maintenance of mitochondrial structure in male animals.

Androgen stimulates mitochondrial biogenesis through activation of the AR/PGC-1α/TFAM pathway (67). PGC-α is a crucial positive regulator of mitochondrial biogenesis, and TFAM plays a vital role in the transcription and replication of mtDNA in mitochondrial biogenesis. When androgen activity is blocked by AR gene knockout, the mitochondrial number is reduced together with a fall in PGC-1α expression in the muscle of ARKO mice (68). The expression of PGC-1α and TFAM in the muscle of the castrated rats (65), mice (69), and pigs (67) was reduced, but these effects were reversed by the administration of exogenous androgen. In the cellular model, testosterone induces the expression of PGC-1α and TFAM in the C2C12 cells (68). AR may mediate the androgen signal through direct binding to the target gene promoter. AREs have been identified in the promoter of TFAM gene (67). However, TFAM seems to exhibit different activities in the muscle cells vs. adipocytes. It was reported that the inactivation of adipocyte TFAM gene protected the knockout mice from obesity and insulin resistance in the dietary obesity model (70). It remains unknown how testosterone regulates PGC-1α expression. Non-genomic pathways may play a role in the regulation, given that no ARE has been identified in PGC-1α gene so far.

Androgens increase mitochondrial content through induction of transcription and duplication of mtDNA, which encodes 13 crucial components of the respiratory chain. Those include the seven subunits (ND1–6 and ND4L) of complex I, CytB of complex III, COX1-3 subunits of complex IV, and two subunits (ATP6 and ATP8) of complex V. Mutations in mtDNA or changes in their copy number are a risk factor for mitochondrial dysfunction, excessive ROS production, and ATP production deficiency, which are often observed in the inherited metabolic diseases (71–73). Castration leads to a reduction in mtDNA copy number (by almost 38%) in the muscle of male pigs (67), suggesting that testosterone is required for the maintenance of mtDNA copy number. Emerging evidence suggests that AR acts in mitochondria to induce gene transcription. AR is found in mitochondria (74), and AREs are identified in the mitochondrial genome (75, 76). AR contains a mitochondrial localization sequence (MLS) for its translocation into mitochondria. Deletion of MLS through gene mutation abolishes AR import into mitochondria (74). Mitochondrial AR is reported in the C2C12 skeletal muscle cell line, with a function similar to the nuclear Ars (77). In prostate cancer cell lines, AR was reported to inhibit respiration chain complex activity in an overexpression study. AR overexpression in PC-3 cells decreases the activity of complex I, complex II, and complex III in the respiratory chain. Inhibition of AR activity by gene knockdown and pharmacological agents increases the complex III activity by 22% and 10%, respectively (74). These results suggest that androgens may regulate the expression and duplication of mtDNA in the control of mitochondrial biogenesis. AR may act directly in mitochondria in addition to its activity in the nucleus. Overactivation of mitochondrial AR may lead to suppression of mitochondrial respiration.

## Testosterone Regulates Mitophagy

Mitophagy determines mitochondrial number and mass. Testosterone inhibits mitophagy as demonstrated by the accelerated mitophagy in the androgen-deficient mice (78). Inhibition of mitophagy was observed by the decreased expression of fusion-control proteins including OPA1 and MFN2 in the castrated rats, which was reversed by androgen supplementation (65, 69). Androgens induced expression of OPA1 in cultured C2C12 cells to promote mitochondria fusion activity (75). In contrast, androgen deficiency increases mitochondrial fission. In the mechanism, an increase in the activity of fission protein DRP1 is observed in cardiomyocytes of castrated rats, which is reversed by androgen supplementation (65). Clearance of damaged mitochondria is enhanced by elevation of LC3 II/I ratio in the castrated mice (79, 80). Digestion of recycled mitochondria requires the fusion of mitophagosome with lysosome, which is promoted by conversion of the inactivated form (LC3-I) into the activated form (LC3-II). An increase in the ratio of LC3 II/I enhances fusion of mitophagosome with lysosome in the skeletal muscle of castrated mice (79, 80), which is observed with reduction in the mitochondrial mass and OXPHOS function. These studies suggest that androgens may raise mitochondrial mass through induction of fusion and inhibition of fission in physiological conditions. In androgen-deficient conditions, this effect is gone, leading to mitochondrial mass reduction through elevated fission and mitophagy.

## Testosterone Affects Mitochondrial ATP Production

Mitochondria are the “powerhouse” in eukaryotic cells to provide energy for cellular activities. ATP production through OXPHOS reactions accounts for about 90% of ATP production in cells (81). Preclinical studies revealed that decreased complex I and II activity was closely associated with decreased ATP production in the cardiomyocytes of obese male rats with insulin resistance (82). Clinical studies revealed that a deficiency of COX in complex IV was associated with decreased ATP production and induced cell apoptosis in the skeletal muscle of humans (83). Additionally, the activity of the ATP synthase β subunit was greatly reduced in the vastus lateralis muscle of obese patients (84). This evidence confirms that deficiency in mitochondrial respiration is closely associated with metabolic disorders.

Androgens are responsible for maintaining the structural integrity of the mitochondrial respiratory chain. A study *in vitro* showed that the AR antagonist, flutamide, decreased complex I activity, mitochondrial membrane potential, and ATP production (by almost 51.2%) in cultured hepatocytes (46, 85). This fall in mitochondrial membrane potential leads to an upregulation of permeability transition pore (PTP) openings, resulting in loss of mitochondrial content and cristae (86). In the hippocampus and substantia nigra of castrated male rats, a lack of androgens is associated with a reduction in ATP synthesis and complex I and complex III activity and a decrease in MFN2 and OPA1 levels in cardiomyocytes (87, 88). Interestingly, mitochondrial dysfunction was reversed by the administration of exogenous testosterone (65, 89). These studies suggest that androgens play a crucial role in the regulation of ATP production through an impact on the mitochondrial respiratory chain. Several mechanisms are discussed below.

Androgens may protect the respiratory chain of mitochondria by alleviating oxidative damage. At physiological levels, ROS serve as a redox messenger in the regulation of multiple cellular processes, including cell growth, differentiation, proliferation, and apoptosis (90). However, excessive ROS cause damage to several biological molecules, including DNA-repair enzymes. Damage to these enzymes consequently causes OXPHOS malfunction (91). Mitochondria are the main site of ROS production; therefore, mtDNA is more susceptible to oxidative damage than nuclear DNA (92). In the skeletal muscle of testosterone-deficient rats, ROS production by mitochondria is increased, as indicated by the increase in plasma malondialdehyde (MDA) concentration (93). ROS increases cell apoptosis in testosterone-deprived men and male rats (94), which were attenuated by exogenous testosterone supplementation (95). Inhibition of AR activity by flutamide also increased ROS (H_2_O_2_) levels and damaged mitochondria, as indicated by a drop in the mitochondrial membrane potential and ATP production in cultured hepatocytes (46). Therefore, these results suggest that androgen/AR may reduce ROS and protect the mitochondrial respiratory chain.

Androgens may also exert their effects through the regulation of cardiolipin, a phospholipid in stabilizing the mitochondrial respiratory chain and IMM structure for mitochondrial function (96). Chemical-induced reduction in testosterone was associated with a significant loss of cardiolipin in the brains of mice (97). However, the connection between cardiolipin and androgens remains to be elucidated.

It should be noted that at least partial effects of testosterone on mitochondria are exerted through its intratissue aromatization, especially in the WAT and brain, leading to the increase in estrogen (98). Aromatase, which is encoded by CYP19A1 gene, actively converts testosterone into estradiol in granulosa cells located in the ovaries (99). Estrogen has a broad impact on energy metabolism through the regulation of mitochondria.

## Effects of Estrogen on Mitochondria

Estrogen is secreted by the ovaries as well as the adrenal gland in females. The active form of estrogen is 17β-estradiol (E_2_). Estrogen typically acts through the nuclear estrogen receptors (ERs) ERα and ERβ. Like androgens, estrogens exert their activity through ER by transcriptional control of gene expression at the estrogen response elements (EREs) on the target genes (100). ERs are activated when a ligand binds to the receptor in the cytoplasm, which is followed by nuclear translocation. ER is also activated by a G protein-coupled receptor, G protein-coupled ER (GPER), which is mainly responsible for rapid non-genomic responses (101).

Estrogen has a profound impact on glucose and lipid metabolism in females. Reduction of estrogen or its receptor is closely associated with impaired energy metabolism, which includes a reduction in lipolysis and glucose uptake and an increased risk of obesity (102). In genetic studies, the inactivation of ERs in the skeletal muscle or the whole body of female mice by ERα gene knockout increased the risk of obesity and insulin resistance (103, 104). In an epidemiology study, the administration of exogenous estrogen reduced the risk of metabolic disorders in postmenopausal women (105). In randomized clinical trials, estrogen hormone therapy greatly reduced fasting glucose, insulin resistance, and the risk of diabetes in postmenopausal women (102). A study done in female mice also confirmed the protective effects of estrogen against obesity and insulin resistance, which disappeared in ovariectomized female mice (106). These studies suggest that estrogen/ERs play a role in the regulation of energy metabolism through effects on glucose and lipid metabolism.

Mitochondria play an important role in estrogen’s effects on energy metabolism (31, 107). In preclinical and clinical studies, reduction in mitochondrial mass and impairment in mitochondrial structure and ATP production are observed in the skeletal muscle of ovariectomized animals or menopausal women (31). Those changes were reversed by the administration of exogenous estrogen. Estrogens affect mitochondria in multiple aspects including protein content and activity, phospholipid content of membranes, oxidant and antioxidant capacities, oxidative phosphorylation, and calcium retention capacities (31). Inactivation of ERα by gene deletion leads to mitochondrial dysfunction and impaired fission–fusion dynamics of mitochondria in females (103). Thus, it is obvious that estrogen has a broad impact on mitochondrial structure and function.

Although less clear than that of ERα in the regulation of energy metabolism, ERβ has also been reported to regulate mitochondrial function (108). ERβ-selective ligands prevented high fat diet-induced lipid accumulation and promoted the expression of mitochondrial biogenesis-related indicators in brown adipose tissue (BAT) and WAT in male and female mice (109, 110). The estrogen-regulated mitochondrial biogenesis markers include PGC-1α (111, 112) and NRFs. Although no ERE has been identified in the PGC-1α gene promoter, it is available in the promoter DNA of NRF-1 gene (113). Estrogen deficiency leads to decreased expression of genes involved in the mitochondrial respiratory chain, oxidative phosphorylation, and metabolic pathways of glucose and lipid in the ovariectomized rats (114–116). This reduction is observed in the transcription factors including NRF-1, TFAM, and PGC-1α in the skeletal muscle of estrogen-deficient female rats (117). These alterations are reversed by the administration of estrogen in ovariectomized rats (118, 119). In addition, estrogen regulates the expression of the COX subunit 7a-related polypeptide (COX7RP), which acts as an assembly-promoting factor for the mitochondrial respiratory chain super complex in the muscle cells (120). COX activity and mitochondrial ATP content are reduced by COX7RP gene knockdown (121). These results suggest that estrogen stimulates mitochondrial biogenesis through the promotion of NRF-1, TFAM, and PGC-1α gene expression, and assembly of the mitochondrial respiratory chain through COX7RP.

Like testosterones, estrogens may act through ERs in mitochondria. The presence of ERs in mitochondria was reported in various cell types by multiple methods, including proteomics analysis of human heart mitochondria, fluorescence probe analysis of human tumor cells, immunoprecipitation of mtDNA, and Western blotting in MCF-7 cells, as recently reviewed (122, 123). ERβ seems to be the main ER present in mitochondria. ERβ exerts several functions by increasing key regulators of mitochondrial function and respiratory chain proteins in cardiomyocytes of female mice, as well as increasing anti-apoptosis effects after pressure overload in the heart tissue of female mice (31).

**Figure 1:**
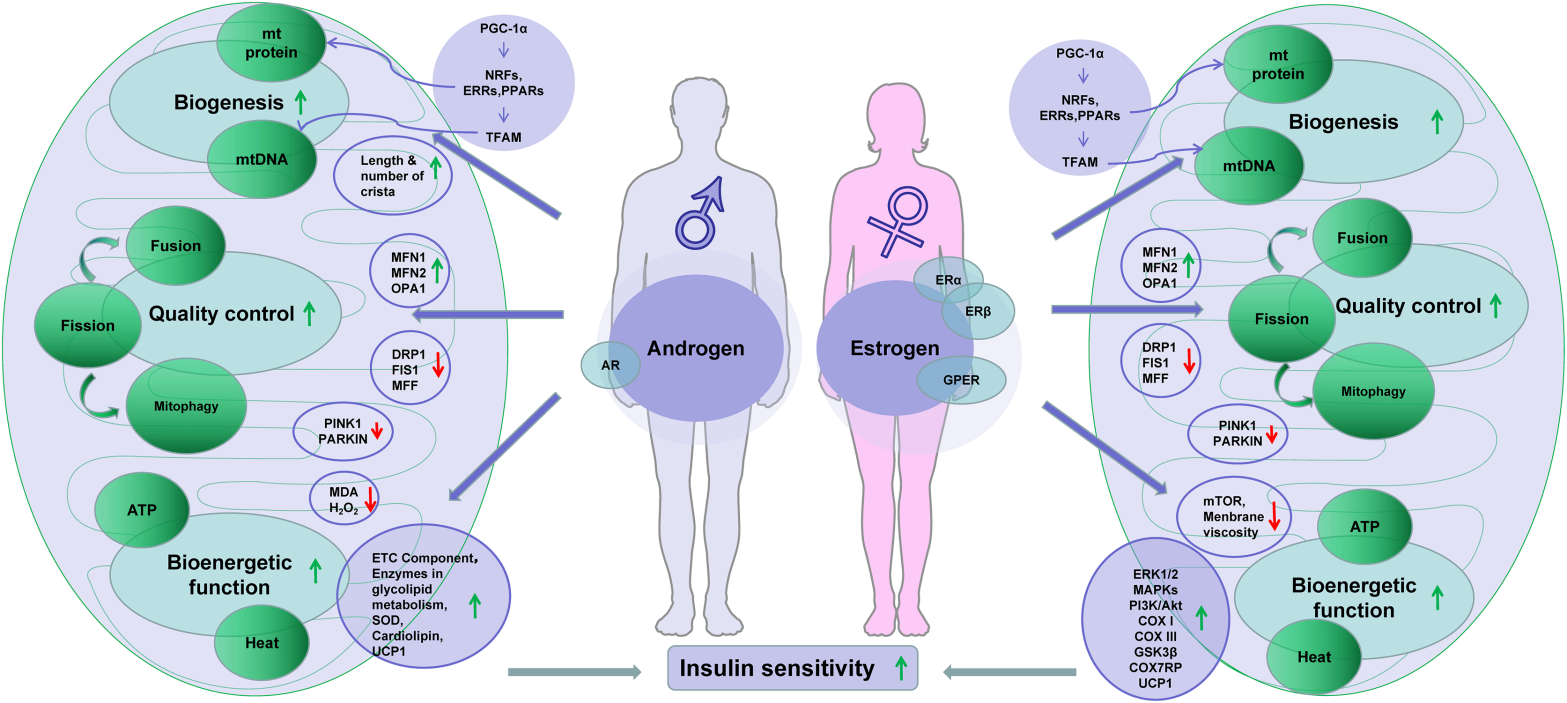
Sex hormones improve insulin sensitivity through regulation of mitochondria. Note: downward arrowheads stand for downregulation, while the upward arrowheads indicate upregulation. Some elements of this figure were produced using Servier Medical Art, https://smart.servier.com.

Activation of ERs may trigger several signaling pathways including extracellular signal-regulated kinase 1 and 2 (ERK1/2), p38 mitogen-activated protein kinases (MAPKs), phosphoinositide 3-kinase (PI3K), c-Jun-NH2-terminal protein kinase (JNK), protein kinase B (PKB), glycogen synthase kinase 3β (GSK3β), β-catenin, calcineurin, and mTOR (124, 125). These activities are reported in the study of ischemia–reperfusion of cardiac remodulating models (126). The ERK, p38 MAPK, and PI3K/Akt signaling pathways are reported to protect mitochondria from H_2_O_2_-induced damage in C2C12 myoblasts (127). In ovariectomized mice, estrogen therapy improved mitochondrial function in the skeletal muscle by correcting membrane viscosity, bioenergetic function, respiration (complex I, III activities), and antioxidant activities (128). Taken together, estrogen plays vital roles in the regulation of energy metabolism through its positive impacts on mitochondrial biogenesis and function. The presence of ERs in mitochondria also makes the effects possible. AR and ERs share location and activities in the mitochondria and nucleus, which suggests a synergy between estrogens and androgens in the regulation of mitochondria (**Figure 1**).

## Excessive Androgen Induces Metabolic Disorders in Female

Androgen and estrogen both promote mitochondrial functions according to the discussion above. Therefore, one may predict that the two hormones may synergize in the regulation of mitochondrial function to control obesity and insulin resistance. However, the fact is opposite to the prediction. Androgen excess is a feature of PCOS in female patients, in which androgen increases the risk for insulin resistance and metabolic syndrome (129, 130). Androgen is elevated in the blood of PCOS patients, which is coupled with higher susceptibility to muscle insulin resistance and obesity (129–131). The mechanism of androgen-related insulin resistance is related to a decrease in expression of adipokines, including adiponectin and omentin-1, which are beneficial to insulin sensitivity in the mouse model of PCOS (132, 133). Other changes associated with androgen excess include an increase in visceral adiposity, reduction of BAT function, and impairment of glucose-stimulated insulin secretion by pancreatic β-cells in PCOS models (61). These factors may partly explain insulin resistance in PCOS, but the basis of those factors remains unknown. We propose that through hormone synergy, androgen may overactivate mitochondria in the presence of estrogen in the female body, leading to insulin resistance.

## Androgen and Estrogen Synergy in Mitochondrial Overactivation

**Figure 2:**
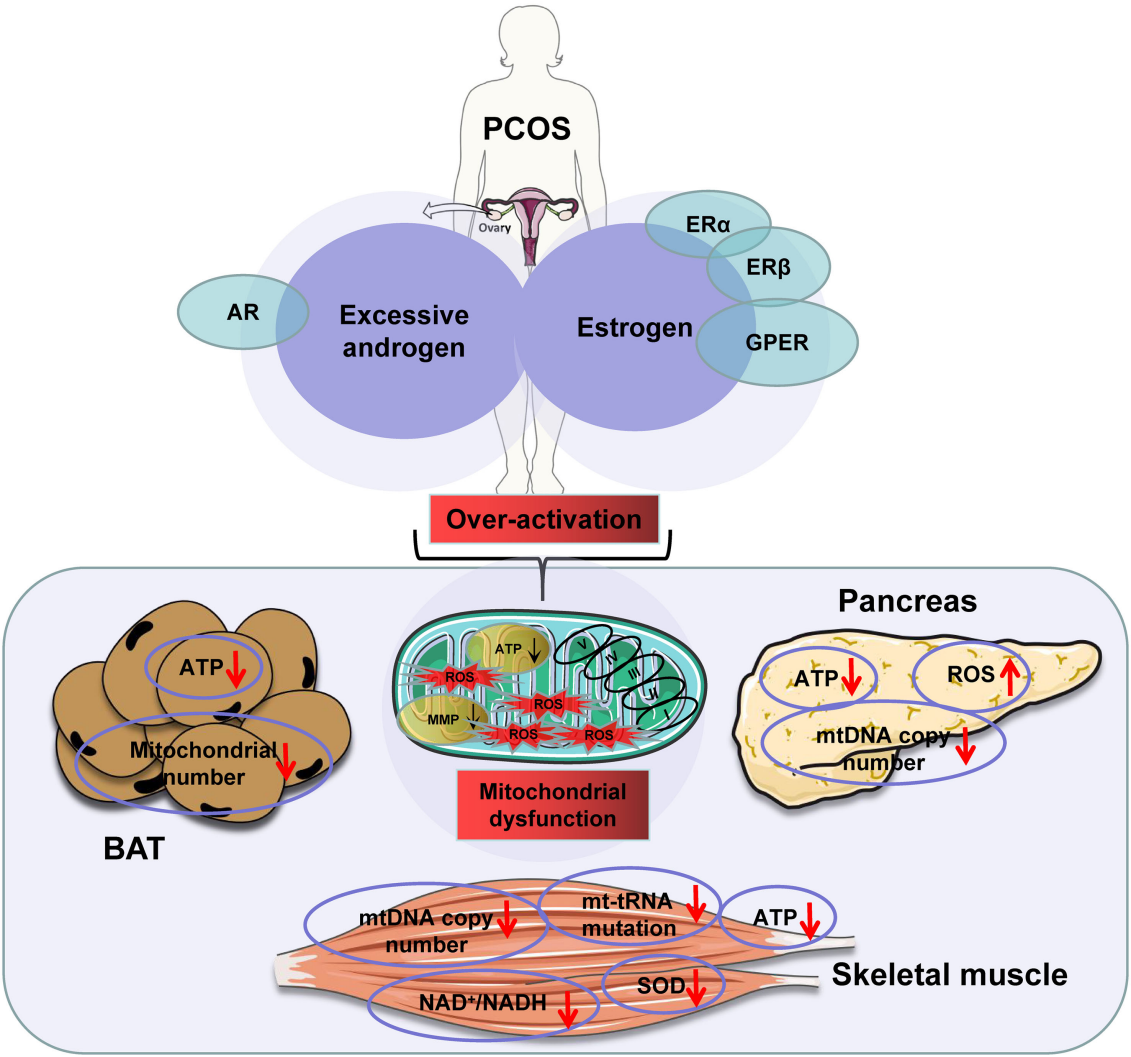
The excessive androgens in the presence of estrogens induce mitochondrial dysfunction in polycystic ovary syndrome (PCOS) patients. Note: downward arrowheads indicate a decrease, upward arrowheads mean an increase. Some elements of this figure were produced using Servier Medical Art, https://smart.servier.com.

ATP production is a primary indicator of mitochondrial function. This function is induced by substrates, hormones, and energy demand as discussed above. In a recent review, excessive substrate availability in obesity has been proposed as a major factor contributing to mitochondrial overproduction of ATP in the mechanism of insulin resistance (134). The insulin resistance occurs following ATP surplus in multiple tissues, in which ATP production exceeds demand (134). ATP surplus in the pancreatic β-cells leads to more secretion of insulin for hyperinsulinemia. ATP surplus in the pancreatic α-cells leads to excessive secretion of glucagon for hyperglucagonemia. These hormone disorders are well-known risk factors for insulin resistance in the muscle, liver, and adipose tissues in obesity. In insulin-sensitive tissues, ATP surplus inhibits the AMPK signaling pathway and activates the mTOR signaling pathway to directly inhibit insulin sensitivity. Additionally, the fall in AMPK activity contributes to mitochondrial degeneration and dysfunction through suppression of mitophagy, which also contributes to insulin resistance. The insulin-sensitizing medicine, metformin, inhibits mitochondrial ATP production in the liver, thereby pharmacologically inducing insulin sensitization. Therefore, ATP surplus due to mitochondrial overactivation is a promising mechanism for insulin resistance. In PCOS, excessive androgen levels may synergize with estrogen to cause mitochondrial overactivation, which in turn leads to mitochondrial dysfunction through the mechanisms discussed above. This possibility is supported by mitochondrial changes in the skeletal muscle, BAT, and β-cells in PCOS models (**Figure 2**).

Mitochondrial dysfunction is a mechanism for muscle insulin resistance in the PCOS models. Mitochondrial dysfunction is associated with insulin resistance in the skeletal muscle in PCOS (135) and in the mouse model of hyperandrogenemia induced by dehydroepiandrosterone (DHEA) injection (136). Mitochondrial dysfunction is reflected by a significant increase in the NAD^+^/NADH ratio and decrease in ATP contents in the PCOS mouse model (137). In patients with PCOS, mitochondrial dysfunction was reported by a reduction in superoxide dismutase levels, mtDNA copy number, mitochondrial membrane potential, and ATP levels (138). However, mitochondrial dysfunction was not observed in the study of PCOS patients using the primary myotubes (139). This dysfunction is likely the result of mitochondrial overactivation by androgens in the presence of estrogens.

The mitochondrial dysfunction is reported in BAT of PCOS models. BAT mitochondria produce heat through UCP1 (140), whose activity is induced by cold temperatures or adrenergic stimulation mimics. Interestingly, BAT mass and function are both decreased in PCOS patients with elevated circulating androgen levels (141). In cellular models, differentiation of brown adipocytes is inhibited by androgen in a dose-dependent manner, leading to decreased expression of UCP1. These changes correspond to a reduction in the mitochondrial number and “whitening” of the interscapular BAT in androgen-induced PCOS models (140). This mitochondrial dysfunction is supported by a decrease in other mitochondrial proteins including PGC-1α and Cidea (cell death-inducing DNA fragmentation factor-like effector A). In addition to direct effects on adipocytes, androgens may affect BAT through rewiring neurons in the central nervous system, which is a proposed mechanism for the central obesity seen in PCOS patients (142).

Androgen effect on β-cells supports that mitochondrion overactivation precedes mitochondrial dysfunction. In a study of the acute effect of androgen on β-cells, androgen was found to induce hypersecretion of insulin through activation of the cAMP/PKA pathway, which was followed by β-cell dysfunction in the female mice (13). In DHT-treated female rats, androgen induced mitochondrial dysfunction in pancreatic β-cells by inhibiting oxygen consumption and ATP production and reducing mtDNA copy number (143). Expression of transcription factors for mitochondrial biogenesis (TFAM, NRF-1, and PGC-1α) was all decreased in the model as well. These observations were confirmed in a later study where higher ADP/ATP ratio, decreased mtDNA copy number, increased ROS production, and downregulation of mitochondrial biogenesis were seen in the β-cells of rat models (144). The exact mechanism behind mitochondrial dysfunction remains unknown. However, overproduction of ROS by mitochondria is a promising theory, as studies have shown that DHT can induce mitochondrial ROS production by altering the balance between oxidative and anti-oxidative arms of mitochondria in PCOS rats (145). As a consequence of ROS elevation, mutations in mtDNA, decreases in mitochondrial membrane potential, and abnormal expression of the respiratory chain complexes were reported in the PCOS rats (146). ROS elevation is associated with a rise in ATP production.

## Mitochondrial Dysfunction Explains Central Obesity in Polycystic Ovary Syndrome Patients

PCOS patients have a high prevalence of central obesity due to increased visceral fat. Accumulation of visceral fat raises the risk of insulin resistance in both men and women (147), especially in postmenopausal female subjects (148–150). Androgen excess is a risk factor for central obesity in females. Inhibition of androgen effects with the receptor antagonist flutamide decreases visceral fat in PCOS patients (151), suggesting a significant role of androgens in central obesity. There are several hypotheses for the effects of androgens on central obesity in literature.

The first is the failure of leptin actions. In PCOS patients, central obesity is associated with a failure of leptin signaling within the central nervous system (142). Leptin conveys the signal of the body’s energy reserves to the central nervous system in the control of food intake and the promotion of energy expenditure. Defects in leptin action may promote central obesity by increasing energy intake and decreasing energy expenditure (152). Exogenous testosterone treatment was reported to reduce serum leptin in humans (153), confirming the regulatory effects of androgen on leptin.

The second is the impairment of new adipocyte generation in the subcutaneous fat. Adipose tissue expansion involves adipogenesis and adipocyte hypertrophy (154). In PCOS patients, subcutaneous fat pads produce androgen, leading to androgen excess (147). Androgens inhibit differentiation of the mesenchymal stem cells into preadipocytes in the subcutaneous fat. This contributes to fat deposition into the visceral fat pads (147).

The third is inhibition of adipocyte lipolysis, thereby promoting hypertrophy of mature adipocytes by androgen. Androgen downregulates signaling pathway proteins important for lipolysis, such as expression of catecholamine receptor and hormone-sensitive lipase (HSL), in white adipocytes (155–157). This decreases fatty acid release from the adipocytes and causes adipocyte hypertrophy. Adipocyte hypertrophy is a risk factor for adipose tissue hypoxia, adipocyte dysfunction, adipose inflammation, and insulin resistance (158, 159).

The fourth is whitening of beige or brown adipocytes following mitochondrial dysfunction to reduce energy expenditure by thermogenesis as discussed above. These studies suggest that androgen excess may promote visceral adiposity through several mechanisms in PCOS patients. Mitochondrial dysfunction appears to be a common player in all four of these mechanisms.

## Conclusion and Perspectives

Sex hormones play a key role in the regulation of energy metabolism. Androgens are required for the maintenance of energy balance in male subjects through the promotion of mitochondrial function. Estrogens have a similar activity in females in the control of energy metabolism through their effects on mitochondria. The activities of these two sex hormones overlap in mitochondria, which suggests a possibility of synergy to induce mitochondrial overactivation in PCOS. These two hormones work through their receptors to induce the expression of nuclear DNA and mitochondrial DNA to promote mitochondrial biogenesis, which lay the foundation for the synergy concept in the induction of mitochondrial function. When the synergistic effect leads to ATP overproduction in cells, it can cause insulin resistance through multiple mechanisms, such as excess secretion of insulin in β-cells and excess glucagon secretion in α-cells. In addition to the endocrine disorders, ATP surplus inhibits AMPK, activates mTOR, and induces ROS production, which contribute to insulin resistance in the skeletal muscle and liver. Mitochondrial overactivation may lead to mitochondrial dysfunction through the alterations of these signaling molecules. Mitochondrial dysfunction in β-cells impairs insulin secretion, which contributes to hyperglycemia in type 2 diabetes. Therefore, mitochondrial overactivation from androgen and estrogen synergism may be a cause of insulin resistance in PCOS.

In the molecular mechanism, the androgen and estrogen synergy may super-induce the activity of PGC-1α because they both upregulate the activity and expression of PGC-1α. Overexpression of PGC-1α is known to induce insulin resistance and impair glucose metabolism in the liver (160). Overexpression of PGC-1α also inhibits glucose-induced insulin secretion in β-cells (161). In addition, the overexpression of PGC-1α induces insulin resistance in the skeletal muscle through the expression of mammalian tribbles homolog (TRB-3) (162), a direct negative regulator of Akt activity in the insulin signaling pathway (163). In type 2 diabetes, chronic hyperglycemia also contributes to the overactivation of mitochondria through the substrate effects (164). This hormone synergy-based view is supported by existing literature on PCOS models and is perfect to fill the gap in the mechanism of metabolic disorder in the PCOS syndrome. However, the possibility remains to be verified by studies *in vivo*. This view represents a unifying mechanism for the distinct roles of androgens in the control of insulin sensitivity in hypogonadal men and PCOS women, which may shed light on a mitochondrion-targeted strategy for the treatment of PCOS in the future.

## Author Contributions

XW and JY contributed to the conception and design of the study. LY wrote the first draft of the manuscript. ML and RW wrote sections of the manuscript. All authors contributed to the article and approved the submitted version.

## Funding

This work was supported by the National Natural Science Foundation of China (NSFC) with a fund (No. 31872801).

## Conflict of Interest

The authors declare that the research was conducted in the absence of any commercial or financial relationships that could be construed as a potential conflict of interest.

## Publisher’s Note

All claims expressed in this article are solely those of the authors and do not necessarily represent those of their affiliated organizations, or those of the publisher, the editors and the reviewers. Any product that may be evaluated in this article, or claim that may be made by its manufacturer, is not guaranteed or endorsed by the publisher.
